# The complete chloroplast genome sequence of *Phlegmariurus carinatus*

**DOI:** 10.1080/23802359.2019.1688720

**Published:** 2019-11-12

**Authors:** Ting Luo, Yunqing Li, Xiaolong Yuan, Yi Wang

**Affiliations:** Laboratory of Forest Plant Cultivation and Utilization, Yunnan Academy of Forestry, Kunming, China

**Keywords:** *Phlegmariurus carinatus*, chloroplast, Illumina sequencing, phylogenetic analysis

## Abstract

The first complete chloroplast genome (cpDNA) sequence of *Phlegmariurus carinatus* was determined from Illumina HiSeq pair-end sequencing data in this study. The cpDNA is 150,349 bp in length, contains a large single-copy region (LSC) of 100,582 bp and a small single-copy region (SSC) of 19,455 bp, which were separated by a pair of inverted repeat (IR) regions of 15,156 bp. The genome contains 120 genes, including 79 protein-coding genes, 8 ribosomal RNA genes, and 33 transfer RNA genes. The overall GC content of the whole genome is 34.0%, and the corresponding values of the LSC, SSC, and IR regions are 31.6%, 30.4%, and 44.2%, respectively. Further phylogenomic analysis showed that *P. carinatus* and *Huperzia serrata* clustered in a clade in family Lycopodiaceae.

*Phlegmariurus carinatus* (Desv.) Ching [synonymous with *Lycopodium carinatum* Desv. ex Poir.] is a representative species of the *Phlegmarirus* genus within Huperzioideae in the family of Lycopodiaceae (Zhang and Kong 2000). *P. carinatus* was used as a traditional herbal medicine in China for long time (Liu et al. 2014). It has significant curative effect on rheumatoid arthritis, rheumatoid arthritis, muscle and bone pain (Zhang et al. 2016). It contains lycopodium alkaloids, triterpenes, phenolic acids, and flavones (Kogure et al. 2016). Therefore, *P. carinatus* has valuable for pharmaceutical applications (Luo et al. 2010). However, there have been no genomic studies on *P. carinatus*.

Herein, we reported and characterized the complete *P. carinatus* plastid genome (MN566837). One *P. carinatus* individual (specimen number: 5309270453) was collected from Cangyuan, Yunnan Province of China (23°16′17″N, 99°1′15″E). The specimen is stored at Yunnan Academy of Forestry Herbarium, Kunming, China and the accession number is YAFM20180401. DNA was extracted from its fresh leaves using DNA Plantzol Reagent (Invitrogen, Carlsbad, CA, USA).

Paired-end reads were sequenced by using Illumina HiSeq system (Illumina, San Diego, CA, USA). In total, about 21.1 million high-quality clean reads were generated with adaptors trimmed. Aligning, assembly, and annotation were conducted by CLC de novo assembler (CLC Bio, Aarhus, Denmark), BLAST, GeSeq (Tillich et al. 2017), and GENEIOUS v 11.0.5 (Biomatters Ltd., Auckland, New Zealand). To confirm the phylogenetic position of *P. carinatus*, the other four species of family *Lycopodiaceae* from NCBI were aligned using MAFFT v.7 (Katoh and Standley 2013) and maximum likelihood (ML) bootstrap analysis was conducted using RAxML (Stamatakis 2006); bootstrap probability values were calculated from 1000 replicates. *Isoetes yunguiensis* (MK047605) and *Isoetes malinverniana* (MH549640) were served as the out-group.

The complete *P. carinatus* plastid genome is a circular DNA molecule with the length of 150,349 bp, contains a large single-copy region (LSC) of 100,582 bp and a small single-copy region (SSC) of 19,455 bp, which were separated by a pair of inverted repeat (IR) regions of 15,156 bp. The overall GC content of the whole genome is 34.0%, and the corresponding values of the LSC, SSC, and IR regions are 31.6%, 30.4%, and 44.2%, respectively. The plastid genome contained 120 genes, including 79 protein-coding genes, 8 ribosomal RNA genes, and 33 transfer RNA genes. Phylogenetic analysis showed that *P. carinatus* and *Huperzia serrata* clustered in a unique clade in family *Lycopodiaceae* (Figure 1). The determination of the complete plastid genome sequences provided new molecular data to illuminate the *Lycopodiaceae* evolution.

**Figure 1.**
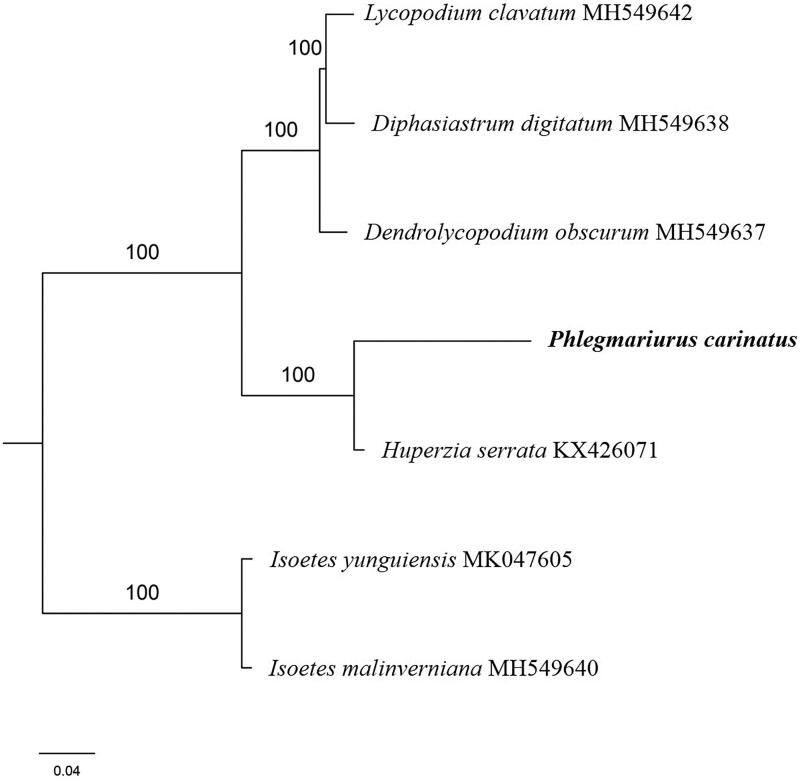
The maximum-likelihood tree based on the five chloroplast genomes of *Lycopodiaceae*. The bootstrap value based on 1000 replicates is shown on each node.

## References

[CIT0001] Katoh K, Standley DM 2013 MAFFT multiple sequence alignment software version 7: improvements in performance and usability. Mol Biol Evol. 30(4):772–780. 23329690 10.1093/molbev/mst010 PMC3603318

[CIT0002] Kogure N, Maruyama M, Wongseripipatana S, Kitajima M, Takayama H 2016 New lycopodine-type alkaloids from *Lycopodium carinatum*. Chem Pharm Bull. 64(7):793–799. 10.1248/cpb.c16-00171 27020466

[CIT0003] Liu F, Liu YC, Jiang WW, He J, Wu XD, Peng LY, Su J, Chen X, Zhao QS 2014 Carinatines A and B, lycopodium alkaloids from *Phlegmariurus carinatus*. Nat Prod Bioprospect. 4(4):221–225. 25089240 10.1007/s13659-014-0030-6 PMC4111872

[CIT0004] Luo HM, Li Y, Sun C, Wu Q, Song JY, Sun YZ, Steinmetz A, Chen SL 2010 Comparison of 454-ESTs from *Huperzia serrata* and *Phlegmariurus carinatus* reveals putative genes involved in lycopodium alkaloid biosynthesis and developmental regulation. BMC Plant Biol. 10(1):209. 20854695 10.1186/1471-2229-10-209 PMC2956558

[CIT0005] Stamatakis A 2006 RAxML-VI-HPC: maximum likelihood-based phylogenetic analyses with thousands of taxa and mixed models. Bioinformatics. 22(21):2688–2690. 16928733 10.1093/bioinformatics/btl446

[CIT0006] Tillich M, Lehwark P, Pellizzer T, Ulbricht-Jones ES, Fischer A, Bock R, Greiner S 2017 GeSeq-versatile and accurate annotation of organelle genomes. Nucleic Acids Res. 45(W1):W6–W11. 28486635 10.1093/nar/gkx391 PMC5570176

[CIT0007] Zhang ZL, Hz L, Guo X, He L, Song JY, Sun C, Luo HM 2016 Cloning and bioinformatics analysis of 1-hydroxy-2-methyl-2-(E)-butenyl 4-diphosphate reductase (PcHDR1) gene in *Phlegmariurus carinatus*. China J Chin Mat Med. 41:4169–4174. 10.4268/cjcmm20162214 28933084

[CIT0008] Zhang LB, Kong XX 2000 Taxonomy of the genus *Huperzia* Bernh. (sen. str.) sect. Serratae (Rothm.) Holub in China. Acta Phytotaxon Sin. 38:13–22.

